# Measurement-Based Modeling of Large-Scale and Time-Varying Small-Scale Fading for LoRa in Indoor Multi-Floor Environments

**DOI:** 10.3390/s26041152

**Published:** 2026-02-10

**Authors:** Gabriel Nascimento Lira, Danilo Brito Teixeira de Almeida, Daniel da Silva Sarmento, João Victor Gadelha Cavalcante Ciraulo, Fabricio Braga Soares de Carvalho, Waslon Terllizzie Araújo Lopes

**Affiliations:** 1 Undergraduate Program in Electrical Engineering (PPgEE), Center of Alternative and Renewable Energies (CEAR), Federal University of Paraíba (UFPB), João Pessoa 58051-900, PB, Brazil; gabrieln.lira@estudante.cear.ufpb.br (G.N.L.); joao.ciraulo@estudante.cear.ufpb.br (J.V.G.C.C.); 2 Post-Graduate Program in Electrical Engineering (PPgEE), Center of Alternative and Renewable Energies (CEAR), Federal University of Paraíba (UFPB), João Pessoa 58051-900, PB, Brazil; danilo.almeida@estudante.cear.ufpb.br (D.B.T.A.); daniel.sarmento@embrapii.cear.ufpb.br (D.S.S.); waslon@cear.ufpb.br (W.T.A.L.)

**Keywords:** smart building, IoT, LoRa, wireless sensor networks, propagation

## Abstract

The deployment of robust Internet of Things (IoT) networks within smart buildings requires a thorough understanding of radio propagation in complex indoor environments. Long Range (LoRa) technology is a promising solution for such applications due to its long range and low power consumption. However, its performance in multi-floor structures is heavily influenced by site-specific propagation conditions. This paper presents an empirical characterization of LoRa signal propagation at 433 MHz within a four-story university building. Extensive measurements of Received Signal Strength Indicator (RSSI) and Signal-to-Noise Ratio (SNR) were conducted to model both large-scale and small-scale fading effects. A log-distance path loss model with a Floor Attenuation Factor (FAF) was derived, yielding a path loss exponent of n=2.53, an FAF of 5.52 dB per floor, and a log-normal shadowing standard deviation of σ=6.93 dB. Time-varying small-scale fading was successfully characterized by a Markov-modulated process (Markov Small-Scale Fading). Furthermore, a non-linear relationship between RSSI and SNR was identified and modeled using a four-parameter logistic function, revealing a dynamic range of approximately 30 dB for the transceivers and a minimum measurable RSSI of −125 dBm. The results validate the proposed models and demonstrate that LoRa can provide reliable, building-wide wireless sensor coverage, offering essential guidelines for the planning and deployment of indoor IoT infrastructure in multi-floor environments.

## 1. Introduction

Digital evolution is happening quickly nowadays, resulting in a significant increase in the number of people connected to the Internet daily. In this context, the interconnection between physical objects, known as the Internet of Things (IoT), has become increasingly prevalent. With the emergence of new technologies, new demands also arise, and consumer behavior patterns tend to adjust to this new dynamic.

Recently, the concept of IoT has helped expand the rise of smart cities [1]. Such a definition is being adopted fast, driven by the growing demand for devices that simplify everyday tasks in a city or urban area, from disabling alarms to automating household chores, including traffic management and air quality monitoring in large cities. When it comes to smart buildings, these demands are directed towards indoor environments [2]. Various communication standards and protocols have emerged with the goal of establishing smart cities and promoting the progressive automation of environmental and urban scenarios. Through sensors and actuators, it is possible to remotely control different variables.

The ways of integrating these devices are varied, including Wi-Fi and Bluetooth. In this strategy of establishing efficient wireless communication, Long Range (LoRa) radio frequency technology stands out, which, unlike Wi-Fi and Bluetooth, offers greater range and low energy consumption. LoRa technology has become prominent in the context of long-distance, low-power networks, being widely used in IoT applications. Due to its robustness and reliability, LoRa has been applied in urban scenarios for environmental monitoring, traffic control, and smart infrastructure [3].

With the growth and consolidation of smart cities, the monitoring of different variables now also includes indoor environments. Consequently, the concept of smart buildings emerged, where sensor networks are installed in enclosed spaces to monitor parameters such as temperature, relative humidity, air quality, luminosity, and many other possible parameters in multi-floor buildings with different structural configurations [4]. With the integration of smart buildings using various sensors, it becomes necessary to integrate these devices so that the measured data can be properly processed and used to optimize the operation of these environments [5,6,7].

As these are multi-floor environments with walls and other obstacles, modeling the wireless channel in smart buildings is challenging. The effects of electromagnetic signal propagation between corridors, walls, floors, stairs, and other scenarios take into account different factors, such as the type of building material, the frequency range of the transmitted signal, as well as the study of various phenomena such as reflection, refraction, dispersion, and shadowing.

To address these aspects, this paper presents a detailed empirical study and modeling framework for LoRa propagation at 433 MHz within a multi-floor university building. The main contributions of this work are threefold:

- We conduct extensive measurements to characterize both large-scale Path-Loss (PL) and time-varying small-scale fading. The large-scale attenuation is modeled using a log-distance model enhanced with a Floor Attenuation Factor (FAF).
- We model the small-scale fading envelope as a Markov-modulated process, named Markov Small-Scale Fading (MSSF), capturing the state-dependent statistical variations caused by human movement and environmental dynamics.
- We identify and model the non-linear relationship between the Received Signal Strength Indicator (RSSI) and the Signal-to-Noise Ratio (SNR) using a four-parameter logistic function, characterizing the practical dynamic range of the LoRa transceivers and enabling more accurate link quality estimation.

The derived models provide a validated framework for simulating and planning reliable LoRa-based WSNs in similar multi-floor indoor environments, directly supporting the design and implementation of future smart building IoT infrastructure.

This paper is organized as follows: Section 2 highlights the state of the art of LoRa applications in different scenarios. Section 3 provides an overview of LoRa technology, covering both its physical layer modulation and the LoRaWAN protocol. Section 4 reviews the fundamental radio propagation concepts relevant to indoor multi-floor environments. Section 5 details the experimental materials and methodology used for data collection. Section 6 presents and discusses the key results, including the characterization of the RSSI–SNR relationship, distance- and floor-dependent performance, path loss modeling, and small-scale fading analysis. Finally, Section 7 summarizes the main conclusions and suggests directions for future work.

## 2. Related Work

There are several relevant publications focusing on IoT and LoRa applications in different scenarios that characterize urban and smart cities environments, including the focus of this work (smart buildings and multi-floor structures).

Smart cities are arising and evolving throughout the world. The authors of Ref. [1] developed a platform in the city of Aveiro, in Portugal, which integrates different solutions and features for all citizens. Energy-saving protocols to route data gathered by distinct sensors widespread in a smart city are investigated by [8]. Replicating the concept of a smart city in a controlled site, leading to a smart campus, enables activities such as counting people in the campus, vehicle monitoring, and vigilance applications based on LoRaWAN [9].

Focusing on smart buildings and their complex environments, new research lines arise. With several potential sensor nodes deployed in a building, the definition of the optimal gateway position (to maximize the performance of the wireless sensor network) requires machine learning and artificial tools, as presented in [5]. With the integration of smart buildings using various sensors, it becomes necessary to integrate these devices so that the measured data can be properly processed and used to optimize the operation of these environments. Not only LoRa is adopted in such environments, but devices based on the IEEE 802.15.4 protocol, such as ZigBee solutions, are evaluated and compared to LoRa, taking into account different technical parameters (bandwidth, range, and battery life) [6,7].

Energy consumption in indoor environments is one of the main challenges for smart building applications–whether it is to maintain the operation of the various devices that integrate and monitor the entire environment, or to optimize the consumption of resources, such as electricity, air conditioning, heating, among other possibilities [2]. Special needs from the users of those constructions are detailed in [10].

The coverage of a LoRa device, and more generally, the performance of a wireless sensor network, is directly affected by several aspects, such as the operation frequency of the device, obstacles, vegetation, and topography. In Ref. [11], the authors investigate LoRa transmission parameters and other aspects that influence the quality of the propagation of the signal in indoor environments. For this goal, a test platform was developed to assess different combinations of the LoRa transmission parameters. A comprehensive study of the LoRa technology in multi-floor buildings (including measurements, characterization, and modeling) is detailed in [4].

Practical aspects related to LoRa networks deployed to support specific industries and other services (as water distribution, for example) also attract the attention of scientists and engineers. In Ref. [12], the reliability of LoRa transmission in Non-Line-of-Sight (NLoS) conditions, as well as in noisy and mobile environments that characterize Industrial IoT (IIoT) applications, are evaluated. A decentralized architecture for industrial automation based on IoT, Lora and Digital Twin (DT) is presented in [13]. The adoption of LoRa networks [14] and hybrid solutions [15] envisage a better distribution of water in different countries.

The native vegetation, present in smart cities and surrounding buildings in large and small cities, inspires the development of new and efficient solutions to protect those environments with the application of technology in environmental monitoring and ecosystem conservation. This is highlighted in several applications that use IoT devices to assist in tasks, such as monitoring isolated and natural areas [16], helping the irrigation [17] and monitoring vegetation areas around electrical networks [18].

The effectiveness of LoRa and wireless sensor networks in natural environments, such as dense and wild forests, demands further study [9]. An experimental work analyzed the performance of LoRa communication in urban and forest areas in different continents (specifically in Brazil and Portugal) [19].

## 3. LoRa^®^ Technology

Currently, technologies such as cellular networks and Wi-Fi are widely used. However they require high bandwidth, power, and have high energy consumption, limiting their application in scenarios that demand energy efficiency [20]. In contrast, LoRa technology stands out for offering a solution that balances low energy consumption with long-range communication, making it attractive for applications that require extensive coverage and continuous operation with limited power sources.

In such a scenario, Low Power Wide-Area Network (LPWAN) is gaining prominence, with LoRa being one of the most relevant technologies. Developed by Semtech Corporation and promoted by the LoRa Alliance, LoRa technology uses wireless radio frequency communication, operating in unlicensed frequency bands of the Industrial, Scientific and Medical (ISM) band. Its main advantage lies in its low power consumption combined with the ability to transmit over long distances, even in environments subject to interference.

Although the terms LoRa and LoRaWAN are often used interchangeably, they have distinct functions. LoRa refers to the physical layer of the technology, responsible for modulating and transmitting signals using a technique called Chirp Spread Spectrum (CSS), which ensures greater range and robustness. On the other side, LoRaWAN is the communication protocol responsible for media access control, security, and traffic management, operating at the Media Access Control (MAC) layer or network layer [3]. The LoRaWAN protocol defines the network architecture, establishing device classes, transmission rules, and security mechanisms, which facilitates interoperability and flexibility in IoT applications. Furthermore, the use of unlicensed bands significantly reduces implementation and operating costs.

The growing adoption of LoRa is evidenced by the presence of 410 million nodes equipped with this technology in 2025 [3], playing a fundamental role in the development of smart cities. In these environments, devices such as smart meters, environmental sensors, vigilance cameras and connected urban infrastructure are deployed on a large scale, promoting greater energy efficiency, resource optimization, and a better quality of life for citizens. The use of LoRa in smart cities also enables sustainable and economical solutions, contributing to the automation of public services and the integrated management of resources [21].

### 3.1. MAC Layer—LoRaWAN Protocol

The LoRaWAN protocol is an essential component of the network layer in LoRa communication systems, responsible for managing communication between LoRa devices. It adopts a star topology, where end devices connect to gateways, which in turn forward data to central servers. This structure provides an efficient and scalable form of communication, essential for IoT applications, where many devices need to connect robustly and with low power consumption [22].

### 3.2. Physical Layer—LoRa Modulation

The physical layer is distinguished primarily by the modulation techniques applied. The CSS technique aims to increase signal range and improve resistance to interference. This characteristic is especially valuable in IoT environments, where communication needs to be robust and efficient, even under weak signal conditions or with many obstacles.

The CSS is based on compressed high-intensity radar pulses (chirps), which are waves with varying frequencies over time. There are two main types of chirps: up-chirps, which occur when the signal frequency increases over time, and down-chirps, in which the frequency decreases. These chirps generate a cyclical shift in frequency, going from the lowest to the highest and vice versa. This frequency-hopping modulation process allows for more efficient communication and is less susceptible to noise or interference from other signals [3].

The spectral spreading level applied to the signal is determined by a parameter defined as the Spreading Factor (SF). LoRa modulation offers six SF levels, ranging from SF7 to SF12. This factor has a direct impact on communication performance, and the higher the SF, the greater the range, as the signal will be spread more widely across the bandwidth. However, this increase in range also results in a decrease in the transmission rate and, consequently, a longer transmission time [3].

The choice of the appropriate spreading factor depends on the operating conditions and the needs of each application. A higher SF offers a more robust signal with greater resistance to interference, making it ideal for longer distances or environments with many obstacles. However, this greater robustness comes at the cost of a lower data rate. On the other hand, a lower SF allows for a higher transmission rate, but resistance to interference and signal range will be more limited [3].

## 4. Radio Propagation Fundamentals

The performance of a LoRa network is influenced by a range of physical environmental phenomena, including obstacles and topographical features. The effects of these factors on signal transmission can be characterized using various statistical and empirical propagation models. Moreover, network performance is also subject to variation due to interference and specific attributes of the LoRa transceivers themselves.

These environmental effects arise from established channel propagation mechanisms. The heterogeneity of real-world environments precludes a simplistic characterization of radio propagation channels. Phenomena such as precipitation or foliage introduce a random attenuation of the signal, known as shadowing, which reduces the average received power. Given its stochastic nature, shadowing is typically modeled using statistical distributions, notably log-normal and gamma [23]. In addition to shadowing, the propagating electromagnetic wave is subject to deterministic PL, a geometric attenuation that increases with the distance between transmitter and receiver.

Collectively, PL and shadowing are termed large-scale fading, as they govern variations in the average received signal power. A distinct phenomenon, small-scale fading, affects instantaneous signal power. It arises from the relative motion of the transmitter and receiver, the presence of moving objects, and the effects of multiple scatters, and is generally characterized by distributions, such as Rayleigh, Rice, η-μ, and κ-μ [24]. For LoRa networks operating in diverse settings, both large-scale and small-scale fading models are critical for accurate link budget analysis and network planning.

### 4.1. Empirical Propagation Models: A Brief Overview

While numerous empirical models exist to predict received power as a function of distance, selecting an appropriate model depends strongly on the environment (indoor/outdoor, urban/rural) and the system’s operational frequency. This subsection reviews common models, grouping them by their primary domain of applicability, to contextualize the choice of model for the present indoor study.

#### 4.1.1. Outdoor and Urban Models

For outdoor scenarios, well-established empirical models are often employed. The Hata-COST231 model, for example, is applicable for PL estimation in urban and suburban environments in the frequency range of 1500–2000 MHz [25]. While influential for macro-cell planning, its frequency range and outdoor assumptions make it unsuitable for the indoor, sub-GHz scenario considered in this work.

#### 4.1.2. Indoor Site-Specific Models

For indoor environments, more detailed models that account for architectural features are available. The COST231 multi-wall model modifies the Free-Space Path-Loss (FSPL) equation to include losses from walls and floors, providing a specialized framework for indoor propagation in the 800–2000 MHz band [26]. Similarly, the Motley–Keenan model explicitly sums the FSPL with attenuation from each wall, floor, and ceiling along the direct path, offering high fidelity when exact building layouts are known [27]. These models require detailed knowledge of the physical structure, which may not be available for a large-scale initial characterization.

#### 4.1.3. General Empirical Models

When detailed structural information is unavailable or when a simpler, more general model is desired, models that parameterize attenuation directly with distance are often used. The α-β-γ (ABG) model presents a practical alternative by relating PL to distance, frequency, and environment type through a small set of parameters [28]. Another widely employed model is the log-distance PL model, which describes PL as increasing logarithmically with distance and is characterized by a path loss exponent *n* that reflects environmental conditions. For applications in multi-floor buildings, it can be extended by incorporating a FAF to account for additional losses from signal penetration through slabs and structural elements between floors [29].

### 4.2. Model Selection for the Present Study

Given the objective of characterizing the large-scale attenuation trend in a multi-floor university building without requiring exhaustive architectural details, the log-distance model extended with a FAF was selected for this work. This approach effectively captures the distance-dependent decay and per-floor penetration loss while remaining tractable for large-scale coverage prediction. The path loss for this extended multi-floor log-distance model is given by

(1) $$PL\left(d\right)=P_{L_{d_{0}}}+10n\log_{10}\left(\frac{d}{d_{0}}\right)+N_{f}\mathrm{FAF},$$

where $P_{L_{d_{0}}}$ denotes the loss at the reference distance $d_{0}$, *n* is the path loss exponent, and $N_{f}\mathrm{FAF}$ represents the cumulative losses introduced by signal penetration through the structures separating $N_{f}$ floors [29].

## 5. Materials and Methods

This section describes the experimental setup and methodology developed to evaluate LoRa module connectivity in an indoor environment. The experimental site, data collection procedures, and measurement layout are detailed.

### 5.1. Experimental Scenario

The experimental tests were conducted in the building of the Center for Alternative and Renewable Energies (CEAR), located on Campus I of the Federal University of Paraíba (UFPB) in João Pessoa, Brazil. The building is 69 m long, 33 m wide, and 20 m high, with four floors containing classrooms and laboratories. The walls are made of concrete, and the ceilings are composed of concrete with polystyrene panels.

This environment was selected due to its predominantly rectilinear infrastructure with long, aligned corridors, which facilitates systematic measurements. The presence of physical obstacles, such as concrete walls and laboratory equipment, allows for the evaluation of LoRa module performance under challenging signal propagation conditions.

**Figure 1:**
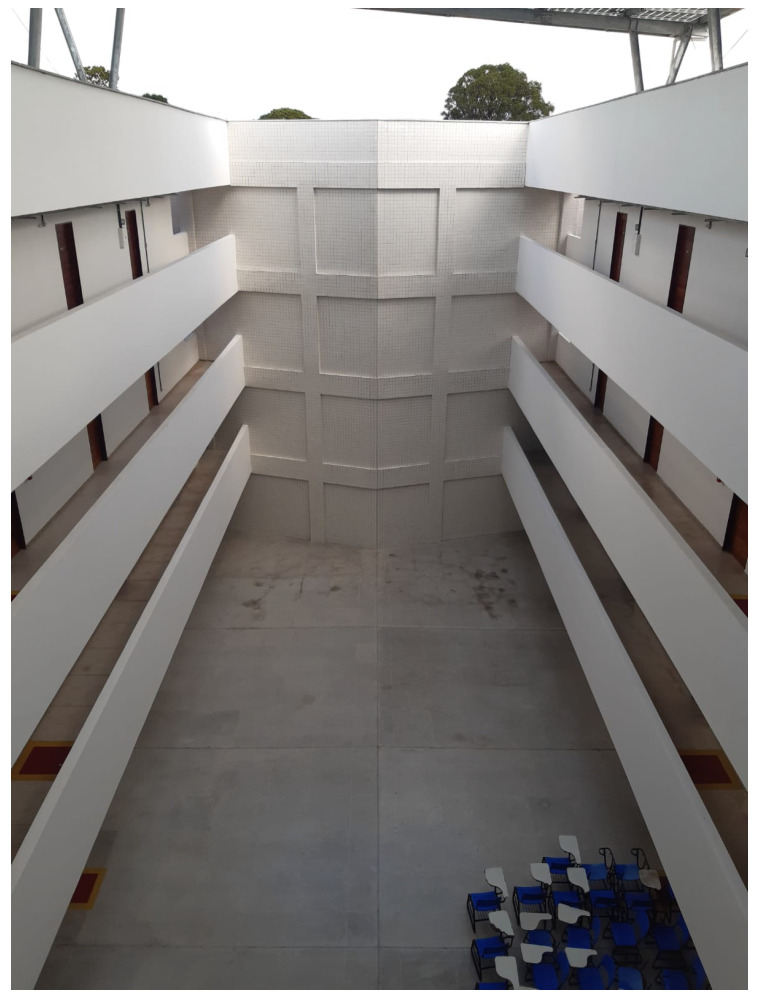
Interior of the CEAR building.

**Figure 2:**
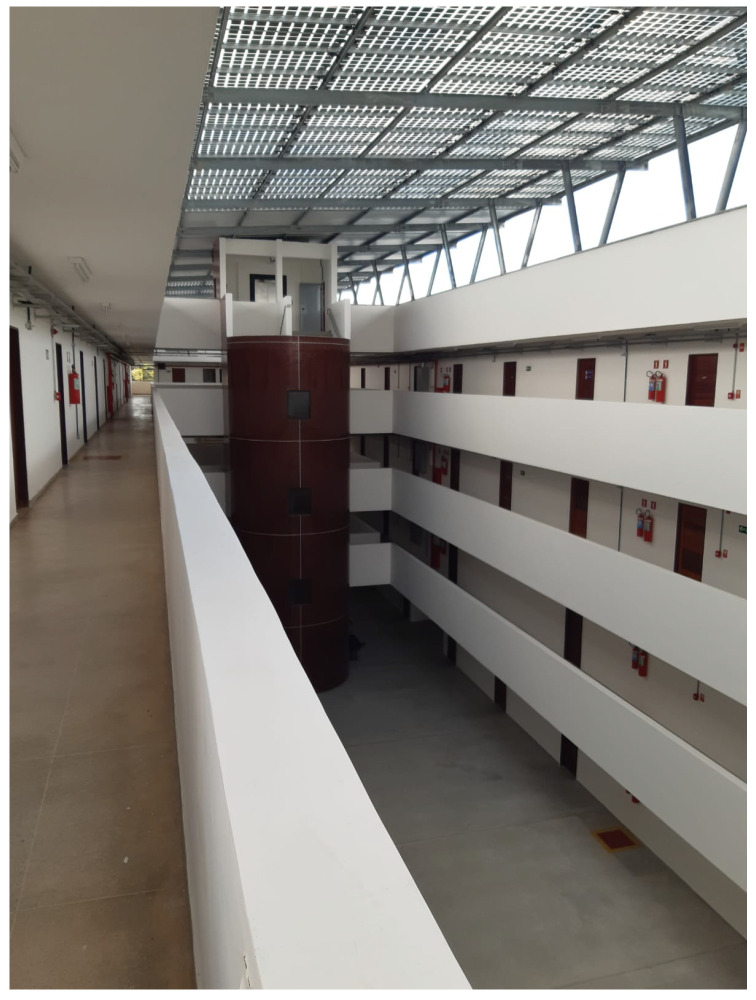
View from the third floor.

Measurements were designed to assess performance degradation as signal propagation complexity increased. Initial measurements were taken on a single floor, with interference primarily from walls and interior objects. Subsequent measurements were performed between different floors to introduce additional attenuation from floor/ceiling structures and more complex equipment layouts. Figure 1 and Figure 2 show the interior corridors, highlighting the structural features and typical obstacles present.

### 5.2. Methodology

The adopted methodology was based on sending packets at 3.5 s intervals from a set of predetermined transmission points. At each point, 500 data packets were transmitted, enabling statistically significant calculation of performance metrics such as SNR and RSSI.

**Figure 3:**
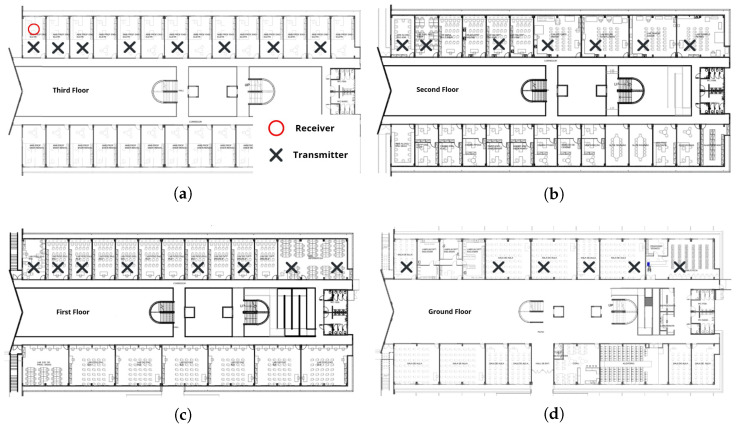
Measurement points in (**a**) the third, (**b**) the second, (**c**) the first, and (**d**) the ground floors.

The receiver’s position was fixed for all measurements in the first room on the third floor, as shown in Figure 3a. The transmitter was sequentially positioned in various rooms and laboratories on different floors, following the points illustrated in Figure 3. Measurements proceeded from the receiver’s location outward, testing connectivity until the link was lost or the building’s physical boundary was reached. This approach allowed for the systematic mapping of the signal’s effective range under varying levels of structural obstruction, both horizontally and vertically.

**Figure 4:**
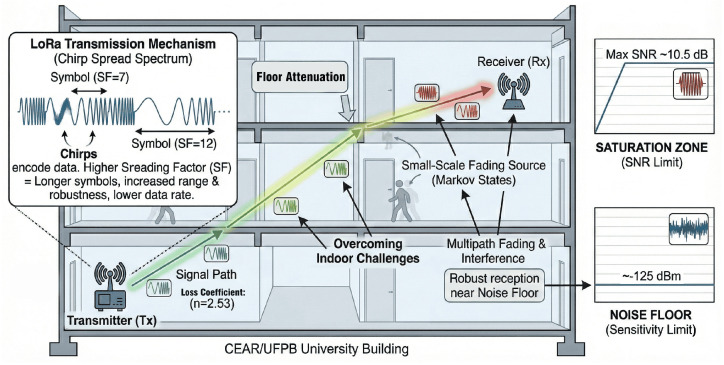
LoRa indoor transmission: chirp spread spectrum and robustness mechanism.

During the measurements in the building, each LoRa packet carried a fixed payload of 32 bytes. The payload size was kept constant in all experiments to ensure a stable transmission time and to isolate the effects of distance and obstacles on the RSSI and SNR values, which were obtained at the receiver. To better contextualize the methodology, Figure 4 illustrates the specific challenges of the indoor environment, such as floor attenuation and multipath fading. It further highlights how LoRa’s Chirp Spread Spectrum (CSS) modulation mitigates these issues to ensure robust reception even close to the noise floor.

### 5.3. Measuring Equipment

This section details the hardware setup used to establish and evaluate the LoRa network. The system comprises two primary modules: a mobile transmitter and a fixed receiver. Their configuration and operational parameters are described below. It is important to note that both modules are identical in their core electronic components for this experiment. The focus was on characterizing the communication link itself, not on sensor data acquisition, which can be integrated into the transmitter as needed for other applications.

#### 5.3.1. Transmission Module

The transmitter unit consists of an Arduino UNO © microcontroller board connected to a LoRa transceiver module. This mobile unit is responsible for broadcasting data packets from various measurement points throughout the building to assess network performance and signal range. It is powered by a portable battery pack to ensure mobility. The transmitted data are received by the fixed receiver and subsequently stored in a database for analysis.

#### 5.3.2. Reception Module

**Figure 5:**
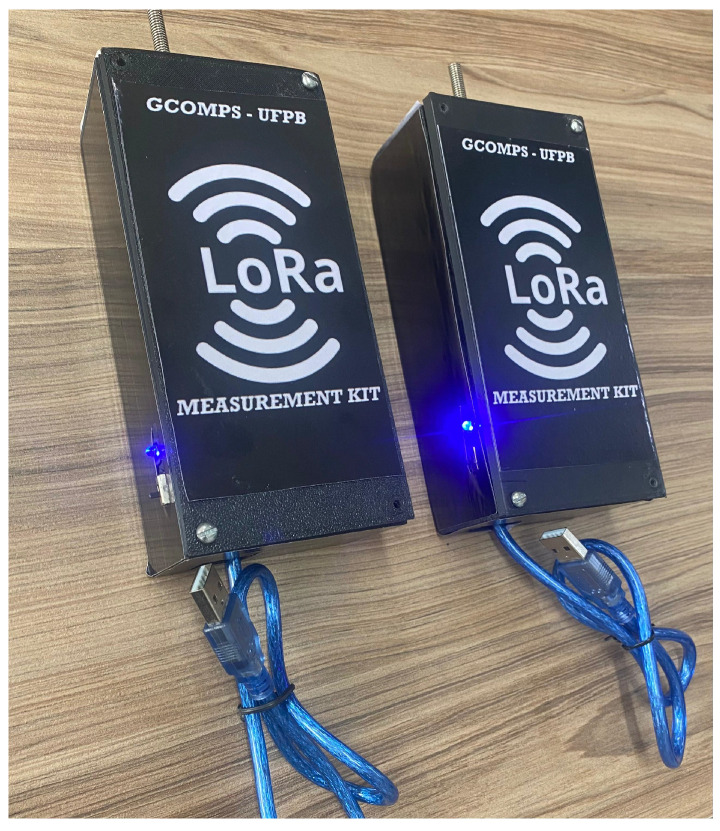
Transmission and reception modules.

The receiver is a stationary unit, also built with an Arduino UNO board and an identical LoRa module. It remains powered via a Universal Serial Bus (USB) connection to a notebook computer. Its function is to receive data packets, log critical link quality metrics, specifically the RSSI and the SNR, and store this information locally. Figure 5 presents the transmission and reception modules developed for this research.

The LoRa modulation parameters considered for these measurements are described in Table 1.

## 6. Results

This section presents and discusses the key findings from the indoor LoRa propagation measurements conducted in the CEAR building. The analysis focuses on the relationship between signal metrics (RSSI and SNR), their dependence on distance and floor separation, the characterization of path loss, and the modeling of small-scale fading as a time-varying phenomenon. The results validate the proposed empirical models against the collected data.

### 6.1. Relationship Between RSSI and SNR

**Figure 6:**
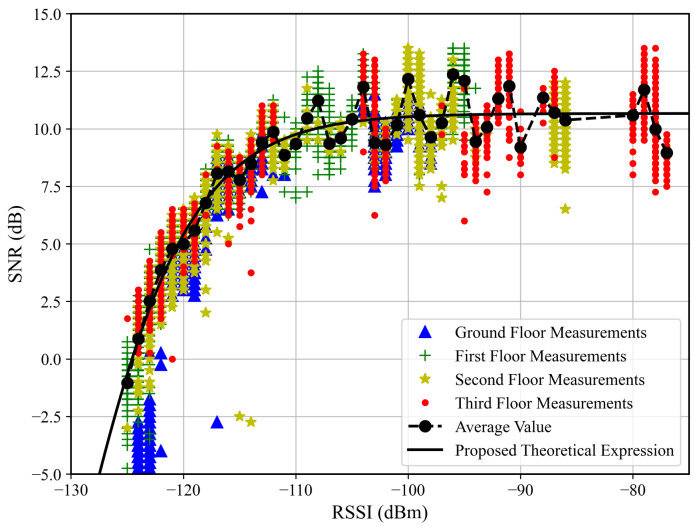
Observed values of SNR as a function of the measured RSSI.

In Figure 6, curves of SNR as a function of the RSSI are presented. The presented data encompass the measurements for different floors and distances between the transmitter and the receiver. The average value curve was calculated by averaging the SNR for each measured RSSI value. A clear trend in the RSSI–SNR behavior is observed, regardless of the floor on which the measurements were taken. However, although the SNR and RSSI are often assumed to have a linear relationship, two saturation regions were perceived. For example, as the RSSI increases, the SNR saturates; additionally, as the SNR decreases, the RSSI saturates. The saturations reveal some aspects of both the building propagation pattern and the adopted LoRa devices’ characteristics.

**Figure 7:**
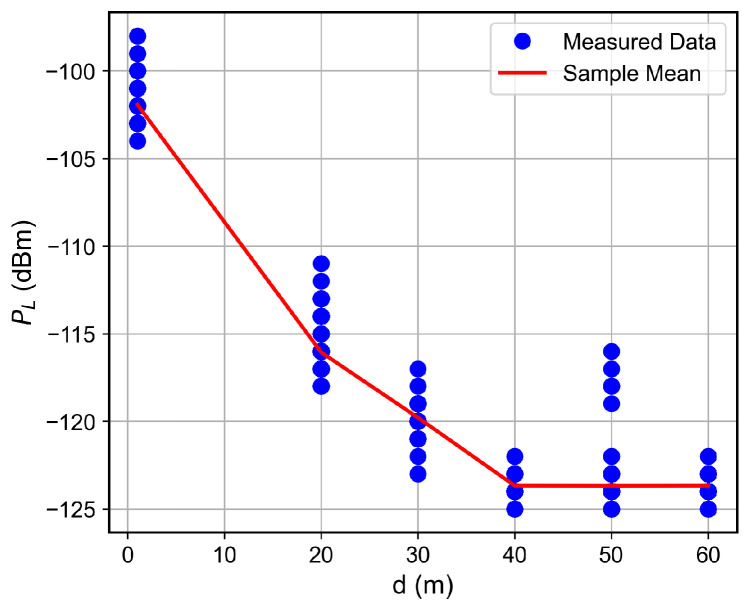
Measured RSSI as a function of the distance for the ground floor.

**Figure 8:**
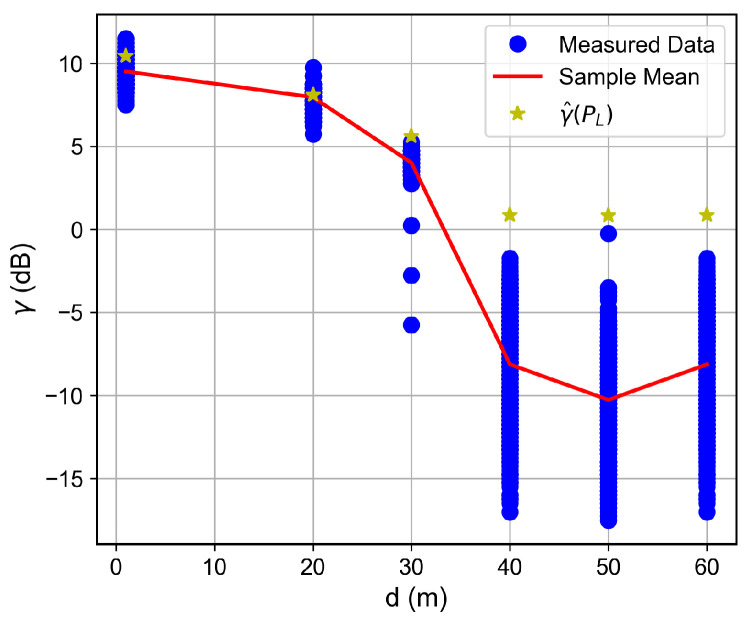
Measured and estimated SNR as a function of the distance for the ground floor.

**Figure 9:**
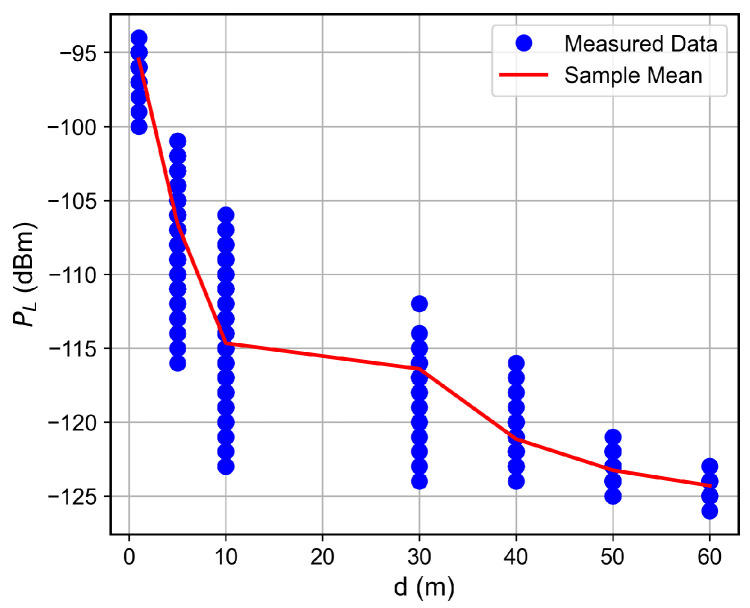
Measured RSSI as a function of the distance for the first floor.

**Figure 10:**
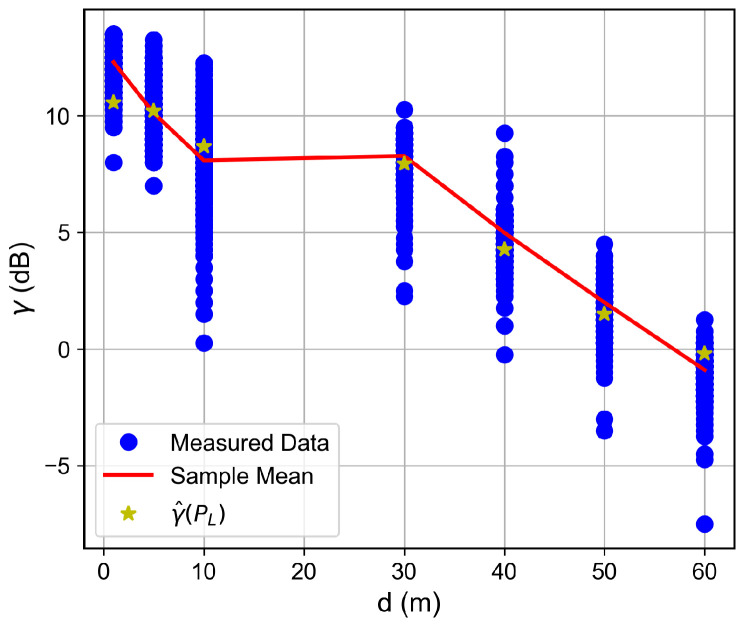
Measured and estimated SNR as a function of the distance for the first floor.

For the low SNR regime, for instance, for SNR below ≈−1 dB, the RSSI keeps fixed as the SNR decreases, which indicates the received signal reached the noise floor level. Therefore, the LoRa device is unable to accurately measure the correct RSSI value. Such a situation was observed for the measurements in which the floor difference or the distance from the receiver to the transmitter was, respectively, greater than or equal to two or greater than 50 m, as observed in Figure 7, Figure 8, Figure 9 and Figure 10. The minimum RSSI value observed was −125 dBm, close to the value reported by the adopted LoRa device’s data sheet [30].

In contrast, as the RSSI increases, the SNR reaches a plateau around ≈10.5 dB. In practice, LoRa receivers have a limited dynamic range, which may not be able to receive strong signals without introducing distortions. Therefore, for high-level reaching signals, if the device performs automatic gain control, both the signal and noise power would be affected, keeping the SNR constant, as observed for RSSI above −100 dBm, implying a dynamic range of ≈30 dB. This observed plateau is therefore a limitation of the practical RF hardware, not a fundamental property of the LoRa modulation scheme.

Based on the measurement results and on the established SNR to RSSI trend, we proposed a four-parameter logistic function to predict the SNR as a function of the RSSI for values that lie inside the adopted LoRa devices’ dynamic ranges. Thus, given the devices and the building’s structures, it is possible to write

(2) $$\begin{array}{c} \hat{\gamma }\left(P_{L}\right)=\gamma_{\min}-\frac{\gamma_{\max}-\gamma_{\min}}{1+e^{-k\left(P_{L}-P_{0}\right)}}, \end{array}$$

where $\hat{\gamma }$ dB is the predicted SNR, $\gamma_{\min}$ dB and $\gamma_{\max}$ dB are, respectively, the SNR at the saturated RSSI region and plateau SNR, $P_{L}$ dBm is the measured RSSI within the device dynamic range, and $P_{0}$ dBm and *k* are shape parameters that control the operating region and the curve slope, respectively. From the measured data, the achieved fit parameters were $\left\{\gamma_{\min},\gamma_{\max},k,P_{0}\right\}=\left\{-35.00,10.66,0.186,-130.97\right\}$.

The SNR is a key LoRa device performance metric. In this way, based on the observed trend and on (2), it is possible to predict the SNR as a function of the distance, given the path loss model. This forward-prediction chain is particularly relevant for network planning. Propagation models estimate the RSSI at various locations, and Equation (2) converts this RSSI into the SNR, allowing identification of areas where the link quality falls below the required threshold. Note that the inverse relationship (RSSI as a function of SNR) is readily derived from Equation (2) and would produce a curve symmetric to the one shown in Figure 6.

Considering the selected LoRa device’s limitation to cover RSSI measurements below −125 dBm, for the processing step, the data acquired for γ≤−2 dB and $P_{L}\leq -125$ dBm were discarded.

### 6.2. Distance and Floor-Dependent Performance

Curves of the RSSI and the SNR measured values, as well as the estimated SNR values obtained from Equation (2), as a function of the distance between the transmitter and the receiver, are shown in Figure 7, Figure 8, Figure 9, Figure 10, Figure 11, Figure 12, Figure 13 and Figure 14.

As expected, both the RSSI and SNR decrease as the distance, and/or the number of floors, between the transmitter and the receiver increases for measurements taken within the dynamic range of the adopted devices. In such situations, the mean value of the proposed $\hat{\gamma }\left(P_{L}\right)$ expression followed the sample mean values closely. However, as the LoRa devices start to operate out of their dynamic ranges, the accuracy of the proposed expression vanishes. It is worth noticing that, outside of the device dynamic range, LoRa is unable to properly measure RSSI and SNR values; thus, as mentioned above, for the processing step, such values must be discarded. It is observed, for instance, that, for d≥40 for the ground floor, the sample RSSI and SNR means are almost constant, reinforcing the need for the adopted data-discarding approach.

### 6.3. Path Loss Modeling

The log-distance path loss model with a FAF was adopted to characterize large-scale fading within the building. This model incorporates floor-induced attenuation via the FAF and captures environmental singularities through an adjustable path loss exponent *n*, offering a more realistic representation than the FSPL model.

For the studied building, the PL expression can be written as

(3) $$\begin{array}{c} P_{L}\left(d\right)=67.71+25.3\log_{10}\left(d\right)+5.52k+\chi_{\sigma }, \end{array}$$

with $P_{L_{d_{0}}}=67.71$ dB, loss coefficient n=2.53, FAF of 5.52 dB, and Log-Normal shadowing with $\chi_{\sigma }$, with σ=6.93 dB.

**Figure 15:**
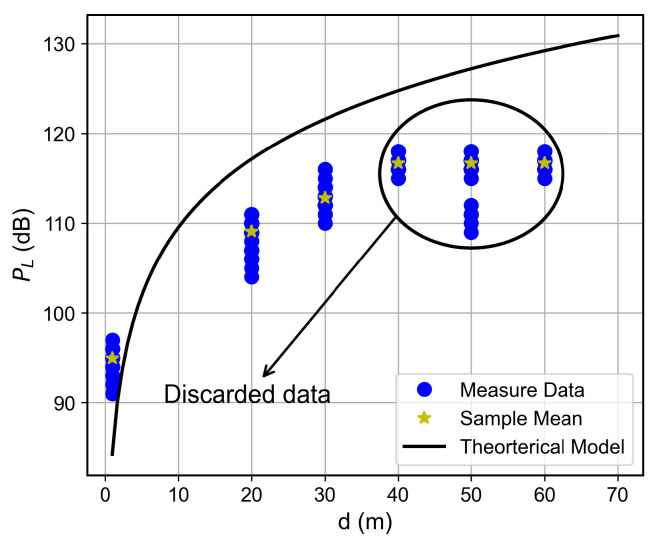
Path loss as a function of the distance for the ground floor.

**Figure 16:**
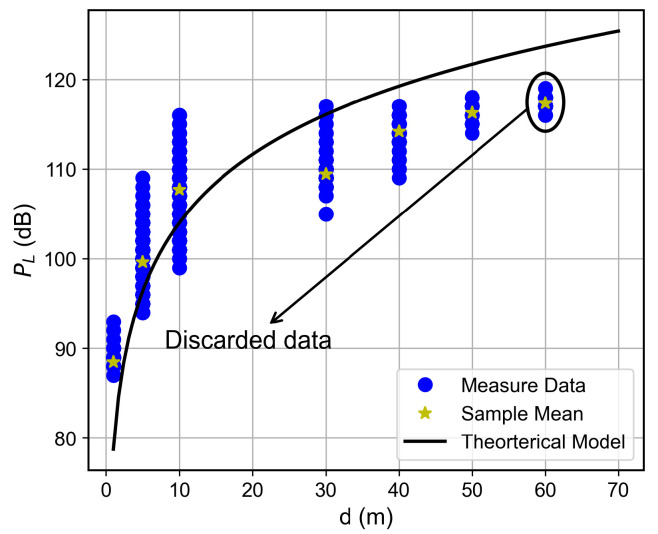
Path loss as a function of the distance for the first floor.

**Figure 17:**
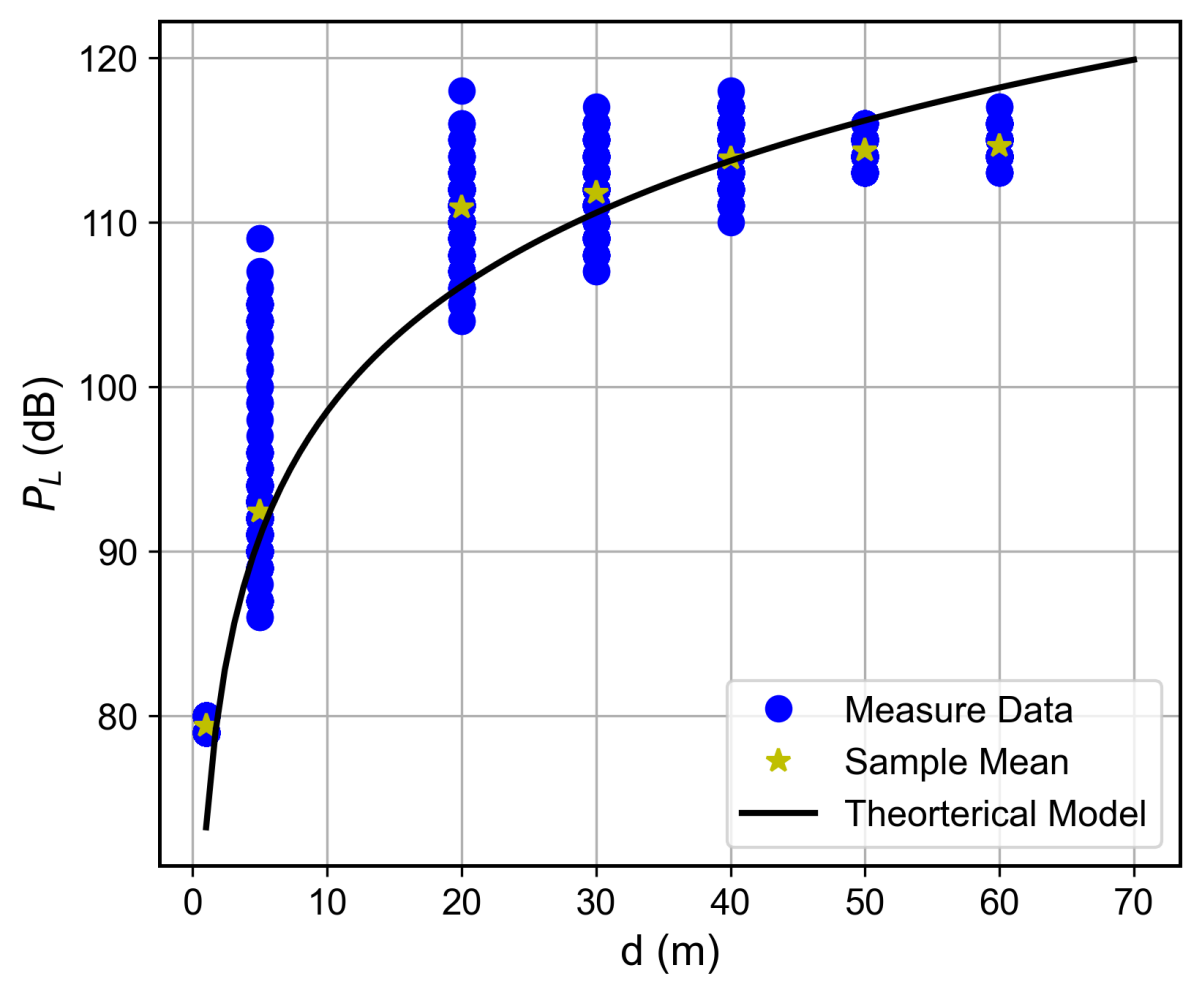
Path loss as a function of the distance for the second floor.

**Figure 18:**
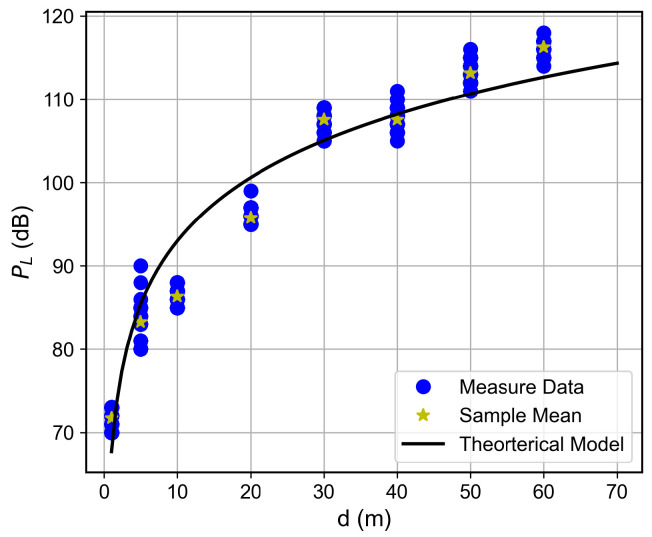
Path loss as a function of the distance for the third floor.

The proposed path loss model, along with the collected data, is presented for each floor in Figure 15, Figure 16, Figure 17 and Figure 18. A good adherence is observed between the model and the measurements’ results, particularly when the number of floors between the transmitter and receiver is low. This trend can be attributed to the increased complexity of the propagation environment with additional floor separations, which introduces more variable obstructions. While the log-normal shadowing component of the model effectively captures the bulk of this variability, the increasing structural complexity naturally results in a wider empirical spread of the measured path loss.

The Motley-Keenan model [27] was also tested for this environment. However, it yielded physically implausible parameters (wall loss ≈2.4 dB and a path loss exponent n<2). This suggests that, at an operational frequency of 433 MHz and given the scale and material properties of the building’s internal structures, the propagating wavefront effectively perceives the medium as quasi-homogeneous. Consequently, it becomes impractical to decouple distance-dependent attenuation from the incremental loss introduced by discrete obstacles, such as individual walls and furniture. This observation reinforces the suitability of the log-distance with FAF approach, which better captures the effective homogenized attenuation of the multi-floor indoor channel.

### 6.4. Small-Scale Fading Characterization

Beyond large-scale fading, which is composed of the PL and the shadowing effect, and affects the signal average power, the channel is affected by the small-scale fading, which acts on the instantaneous channel gain. However, due to the presence of moving people and objects, a time-varying normalized channel gain distribution may be expected. This class of processes can be characterized using a Finite-State Markov Chain (FSMC), in which each state is governed by a different distribution. This process, referred to as the MSSF, has the Probability Density Function (PDF) written in the form of a mixture process, suitable to describe peaky heavy-tailed or multi-modal distributions, poorly characterized by single distributions [31].

To fully describe the MSSF, the state transition matrix, which allows one to derive the steady-state probabilities, and the average state length of stays must be known. So, if for a *N* states MSSF, one defines the steady-state probability vector $\pi =\left[\pi_{1},\ldots ,\pi_{N}\right]={\mathrm{row}}_{N}\left(\Pi^{\infty }\right)$, in which Π denotes the state transition matrix, and the average state length of stays vector $\tau =\left[\tau_{1},\ldots ,\tau_{N}\right]$, the PDF of a MSSF can be written as follows [31]:

(4) $$\begin{array}{c} p_{H}\left(h\right)=\sum_{n=1}^{N}w_{n}p_{H_{n}}\left(h\right), \end{array}$$

in which $w=\left[w_{1},\cdots ,w_{N}\right]=\frac{\pi \tau^{T}}{\mathrm{Tr}\left(\pi^{T}\tau \right)}$ represents the occurrence probability, and controls the influence of the *n*-th state onto the MSSF, and $p_{H_{n}}\left(h\right)$ denotes the *n*-th state distribution.

**Figure 19:**
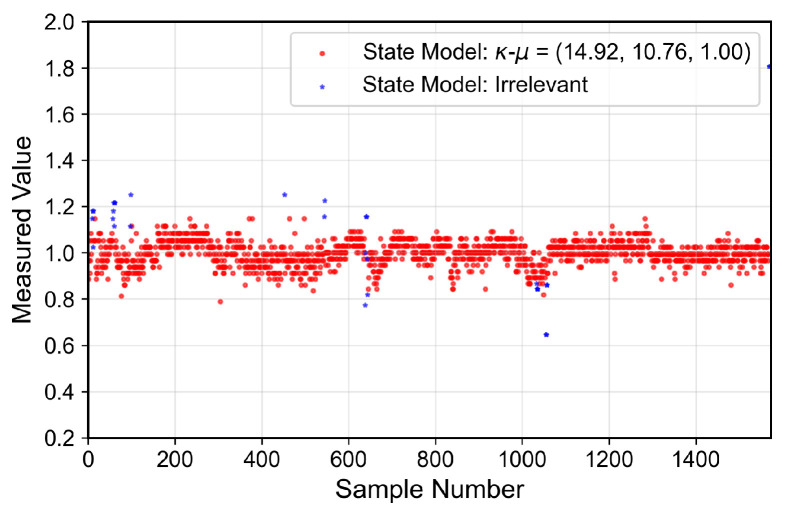
Temporal series of the measured normalized channel gain for the ground floor.

**Figure 20:**
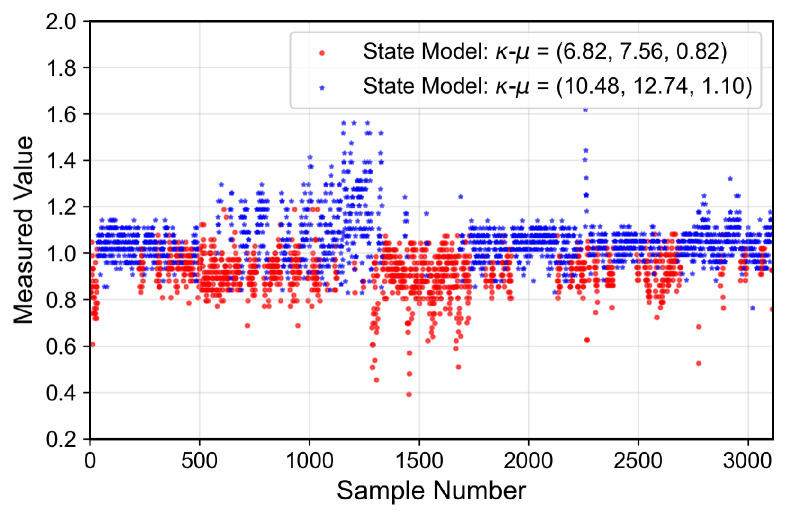
Temporal series of the measured normalized channel gain for the first floor.

**Figure 21:**
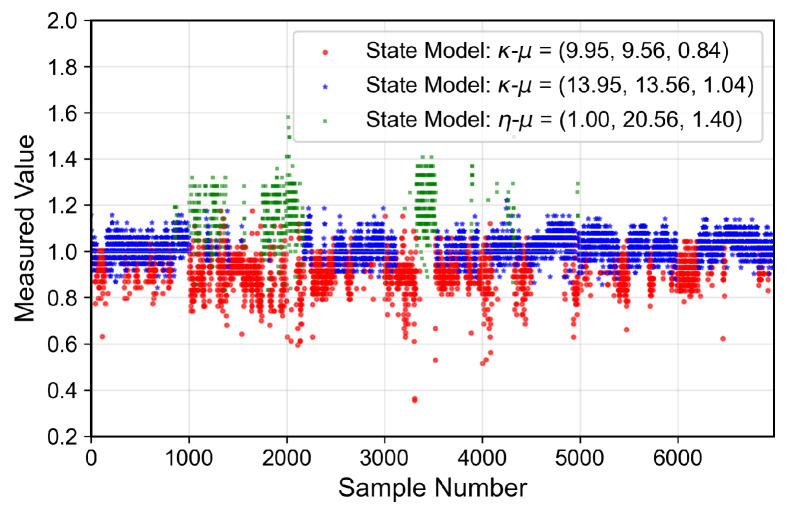
Temporal series of the measured normalized channel gain for the second floor.

**Figure 22:**
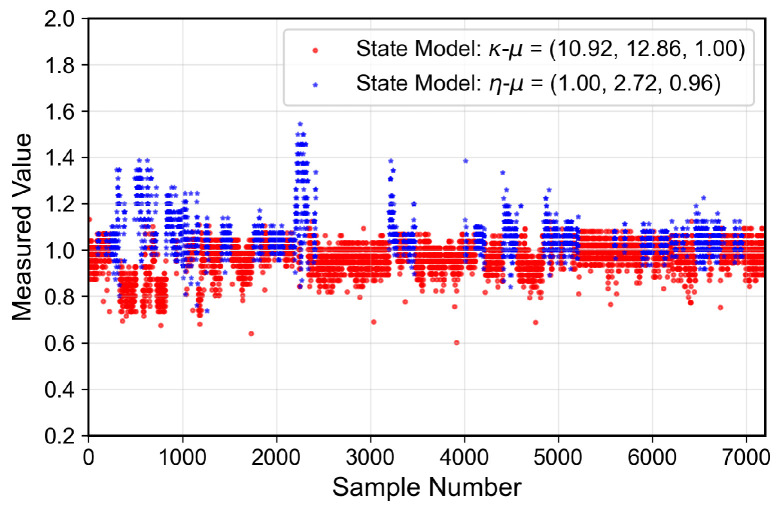
Temporal series of the measured normalized channel gain for the third floor.

The analyzed indoor channel exhibits a multi-mode propagation characteristic, as can be seen in Figure 19, Figure 20, Figure 21 and Figure 22, a suitable candidate for modeling using the MSSF. Furthermore, by characterizing each state by means of generalized distributions, such as κ-μ and η-μ, the proposed approach can cover several distinct scenarios, as these distributions encompass simpler models, such as Rayleigh, Nakagami-*m*, and Rice as particular cases [24].

From Figure 19, Figure 20, Figure 21 and Figure 22, it is observed that, for the first and second floors, the channel gain presents higher variations compared to the ground and third floors. The stability in the ground-floor channel gain behavior may be explained either by the low presence of people within the building during ground-floor measurements or due to the removed data whose measurements were out of the LoRa dynamic range. For example, for the ground-floor, the state changes rarely occurred, allowing a single distribution characterization.

For the distribution parameters estimation step, each distribution was treated separately, based on the samples clustered for each state. Furthermore, the final PDF was composed based on the distribution estimated parameters and their occurrence probability, according to Equation (4).

**Figure 23:**
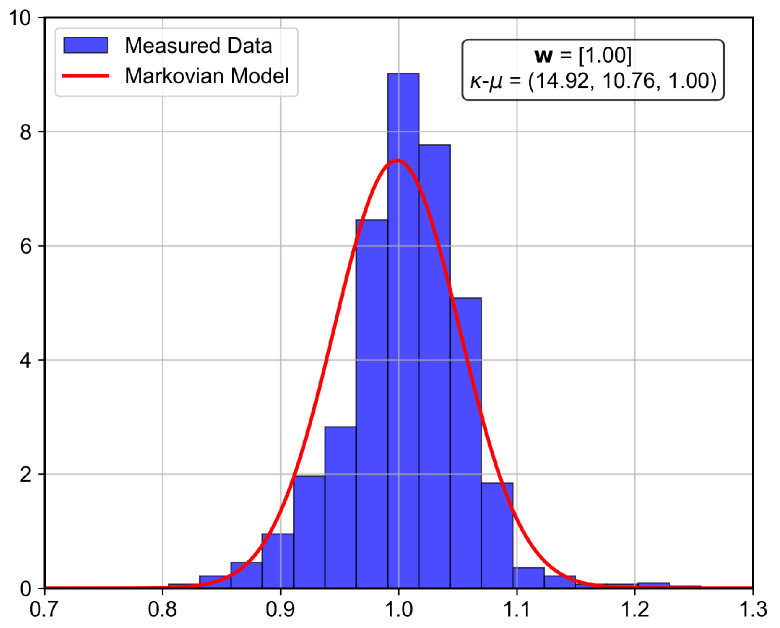
Histogram of the measured normalized channel gain for the ground floor.

**Figure 24:**
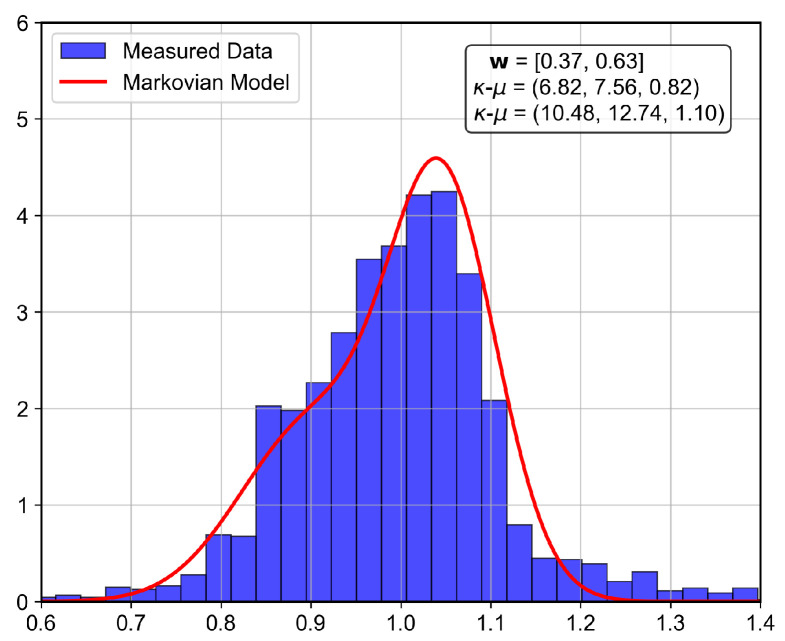
Histogram of the measured normalized channel gain for the first floor.

**Figure 25:**
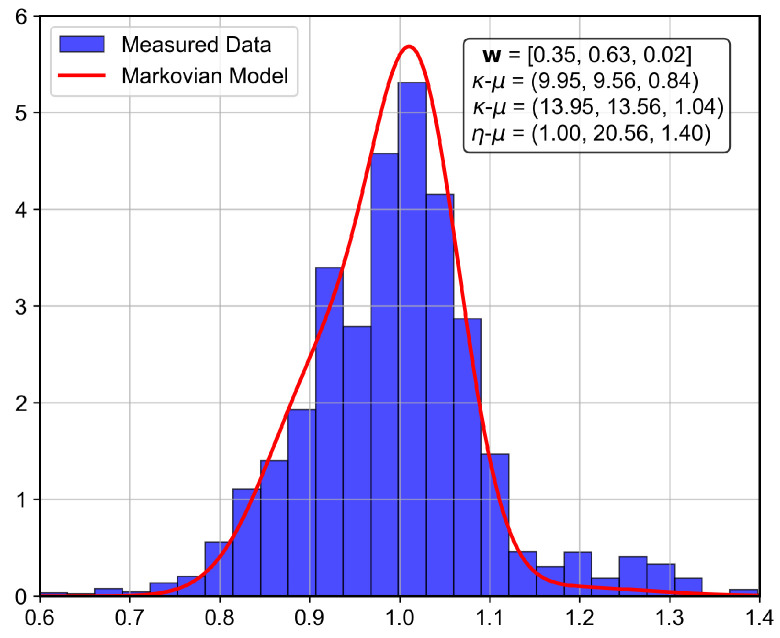
Histogram of the measured normalized channel gain for the second floor.

**Figure 26:**
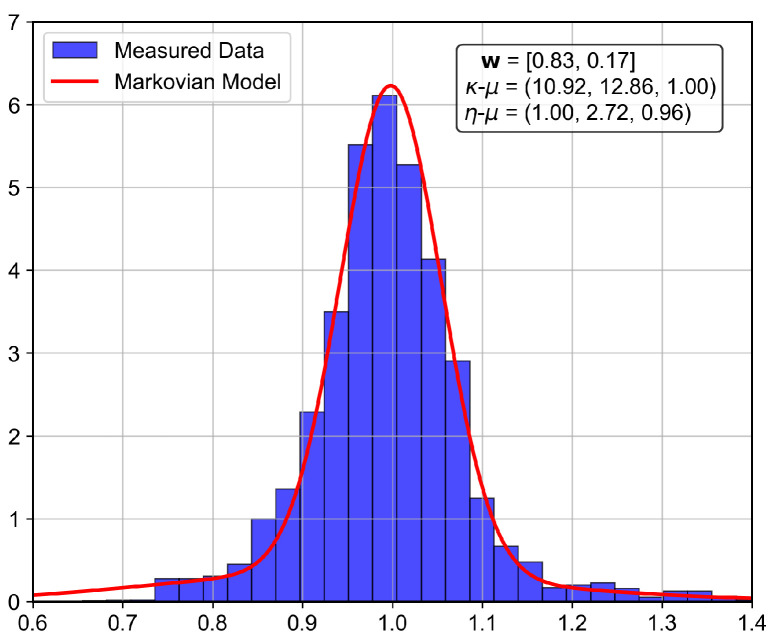
Histogram of the measured normalized channel gain for the third floor.

The estimated state transition matrix and occurrence probability are

$$\Pi_{\mathrm{Grd}}=\left[\begin{array}{cc} 0.992 & 0.008\\ 0.279 & 0.721 \end{array}\right]\;\mathrm{and}\;w_{\mathrm{Grd}}=\left[\begin{array}{cc} 0.999 & 0.001 \end{array}\right],$$

for the ground floor,

$$\Pi_{1\mathrm{st}}=\left[\begin{array}{cc} 0.955 & 0.045\\ 0.035 & 0.965 \end{array}\right]\;\mathrm{and}\;w_{1\mathrm{st}}=\left[\begin{array}{cc} 0.368 & 0.632 \end{array}\right],$$

for the first floor

$$\Pi_{2\mathrm{nd}}=\left[\begin{array}{ccc} 0.948 & 0.032 & 0.020\\ 0.024 & 0.974 & 0.002\\ 0.076 & 0.015 & 0.909 \end{array}\right]\;\mathrm{and}\;w_{2\mathrm{nd}}=\left[\begin{array}{ccc} 0.345 & 0.632 & 0.023 \end{array}\right],$$

for the second floor, and

$$\Pi_{3\mathrm{rd}}=\left[\begin{array}{cc} 0.987 & 0.013\\ 0.028 & 0.972 \end{array}\right]\;\mathrm{and}\;w_{3\mathrm{rd}}=\left[\begin{array}{cc} 0.830 & 0.170 \end{array}\right],$$

for the third floor. Furthermore, the theoretically proposed distribution PDF and the histogram of the measured data are presented in Figure 23, Figure 24, Figure 25 and Figure 26 for the ground, first, second and third floors, respectively.

As expected from the data observed in Figure 19, a single distribution must be enough to characterize the fading envelope of the ground-floor. In addition, the heavy-tail distribution of the data from the third floor and the multi-modal characteristics exhibited by the distribution of the data from the first and second floors reveal that the MSSF is a good choice to characterize such environments.

Both κ-μ and η-μ distributions were employed in the characterization of the MSSF states. The analysis revealed consistently high estimated values for the μ parameter across states. In this high-μ regime, the distribution behavior converges, effectively suppressing the influence of the κ and η parameters, leading to mild, non-severe fading envelopes, making the specific choice between κ-μ and η-μ less critical for the analyzed scenario.

From the estimated distribution parameters, it is observed that all scenarios are scatterer-rich, indicated by the presence of at least one state with a high estimated value of μ. The increase in the μ parameters, specifically, tightens the lobe of the PDF and indicates scenarios with favorable propagation characteristics. Therefore, it is possible to state that the analyzed scenario suffers more from the PL and shadowing than the small-scale fading effect.

While the empirical data were collected at 433 MHz, the derived models have clear implications for other LoRa bands, for example, 868 MHz and 915 MHz. The path loss exponent *n* and FAF, governed by the building’s geometry and materials, are expected to remain similar across these sub-GHz frequencies. However, higher frequencies will experience greater free-space loss and potentially higher attenuation through obstacles, reducing the effective range. The small-scale fading, governed by multipath scattering, may also exhibit richer dynamics due to the shorter wavelength.

### 6.5. LoRa Performance Evaluation

While the preceding sections characterized the propagation channel through metrics like RSSI, SNR, and statistical fading models, it is crucial to evaluate the ultimate impact of these channel conditions on communication link reliability from a system perspective. In this subsection, we assess the performance of LoRa technology in the measured multi-floor environment by analyzing the Packet Loss Rate (PLR) across different SF and Code Rate (CR) configurations. In this context, PLR is defined as the probability that a transmitted packet is either not received or is received with uncorrectable errors, representing the effective packet delivery failure rate that would be experienced by an application layer.

To perform this evaluation, the CRC feature on the LoRa transceivers was disabled for a dedicated set of experiments. This prevented the hardware from automatically discarding corrupted packets, allowing it to capture all received data regardless of error conditions. Each transmitted packet contained a 4-byte sequence number followed by a 32-byte payload generated pseudo-randomly from that sequence number using a deterministic Linear-Feedback Shift Register (LFSR) algorithm. At the receiver, the expected payload was regenerated using the same algorithm and the received sequence number. The received payload was then compared bit-by-bit against the expected reference. A packet was classified as effectively “lost” if either (1) it was not received at all (detected through sequence number gaps), or (2) it was received but contained any bit mismatch with the expected payload (which would cause CRC failure in normal operation). The PLR was calculated as the ratio of lost packets to the total number of packets transmitted. Measurements were performed at a fixed horizontal separation distance of 30 m for communication between different floors.

This methodology provides a realistic assessment of link reliability from an application perspective, capturing both complete transmission failures and corrupted receptions that would be unusable in practice. Since CRC was disabled for measurement purposes, no forward error correction or automatic packet rejection was performed by the hardware during these tests. The PLR results can be directly related to the path loss and SNR predictions from the models developed in previous sections, establishing a comprehensive framework for predicting link reliability based on spatial location and floor separation.

**Figure 27:**
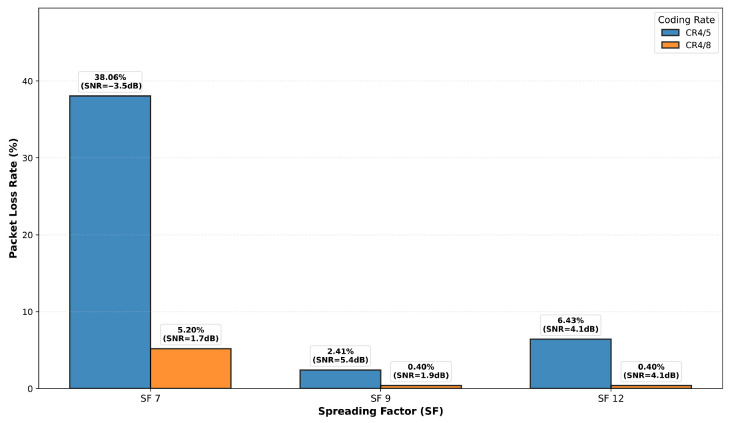
PLR for different CRs and SFs observed in the ground floor.

**Figure 28:**
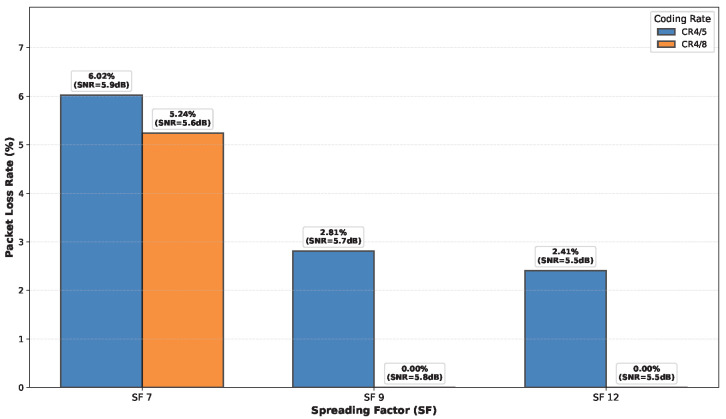
PLR for different CRs and SFs observed in the first floor.

**Figure 29:**
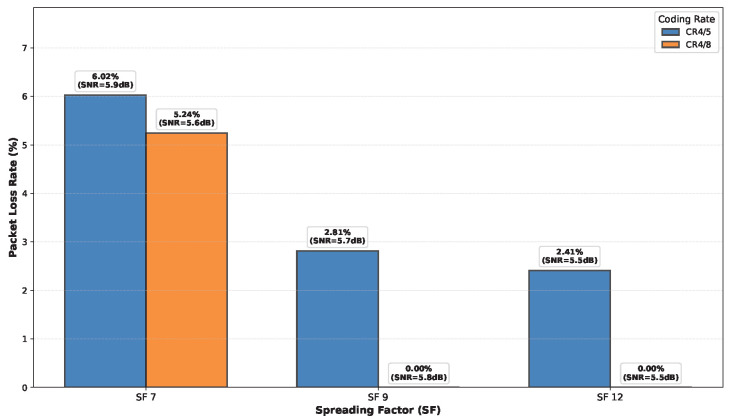
PLR for different CRs and SFs observed in the second floor.

**Figure 30:**
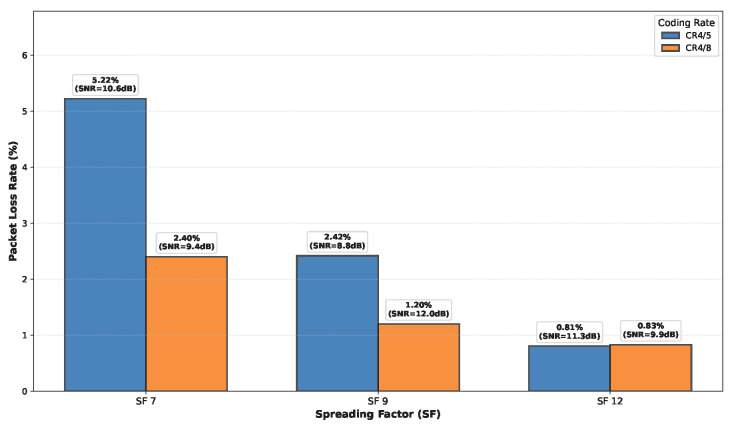
PLR for different CRs and SFs observed in the third floor.

The experimental PLR values for different floor separations at 30 m horizontal distance, for various CRs and SFs are presented in Figure 27, Figure 28, Figure 29 and Figure 30. Three key observations emerge: (1) As expected, higher SF values (SF9, SF12) consistently outperform SF7 across all floor separations due to increased processing gain and receiver sensitivity. (2) More robust coding (CR 4/8) significantly reduces PLR compared to CR 4/5, particularly for challenging cross-floor links where forward error correction provides substantial benefit. (3) The performance degradation with increasing floor separation follows the Floor Attenuation Factor (FAF) model established in Section 6.3, with ground-to-third-floor links showing the highest PLR values. Notably, SF7 with CR 4/5 represents the most challenging operational condition, with PLR reaching 38% for three floor separation communication, while SF12 with CR 4/8 maintains reliable links (PLR < 1%) even across multiple floors. These results provide concrete guidance for selecting appropriate LoRa configuration parameters based on deployment requirements in multi-floor smart buildings.

While the path loss model presented in Section 6.3 provides the mean attenuation, individual measurements exhibit variability due to log-normal shadowing with standard deviation σ=6.93 dB. The RSSI values reported in Table 2 for the performance evaluation campaign show deviations from the model predictions that are consistent with this shadowing distribution. As expected for a physical channel characteristic, the measured RSSI values show no systematic dependence on the LoRa configuration parameters (SF and CR), since path loss is determined solely by the propagation environment and distance. Minor variations between measurements with different SF/CR configurations at the same location fall within the expected statistical range of the shadowing component. For instance, ground floor measurements at 30 m distance show RSSI values ranging from −112 dBm to −109 dBm across different SF/CR combinations, compared to the model prediction of approximately −119 dBm. This 7–10 dB variation is consistent with the σ=6.93 dB shadowing distribution, confirming the statistical nature of indoor propagation and validating the independence of path loss from communication system parameters.

## 7. Conclusions

This work presented a comprehensive characterization of an indoor radio propagation channel based on empirical data acquired using Long Range (LoRa) devices operating at 433 MHz. The analysis encompassed both large- and small-scale fading phenomena. It was observed that, due to the movement of people and objects, the small-scale fading exhibits different distributions over time, with transitions that can be characterized by Finite-State Markov Chain (FSMC) processes. Furthermore, within each state, the small-scale fading was characterized by κ-μ or η-μ distributions. The high number of scatters, characterized by the high observed values of the μ parameter, in most cases μ>7.5, is related to narrow-lobe fading distributions, associated with favorable propagation scenarios. Therefore, it was possible to state that the channel limitations were imposed by the average power attenuation caused by the Path-Loss (PL) and shadowing effects. To characterize the large-scale fading, the log-distance with Floor Attenuation Factor (FAF) model and Log-Normal shadowing were adopted, yielding a loss coefficient of n=2.53, a FAF of 5.52 dB per floor, and a shadow standard deviation of σ=6.93 dB, revealing a good agreement with the collected data, especially when the number of floors between the transmitter and the receiver is lower. Additionally, it was possible to propose a four-parameter logistic function to describe the Signal-to-Noise Ratio (SNR) to Received Signal Strength Indicator (RSSI) relationship, which revealed the dynamic range of the adopted devices. In summary, this study provides validated models for both macroscopic (path-loss) and microscopic (fading dynamics) propagation effects in an indoor LoRa network. The proposed Markov-based small-scale fading model and the modified log-distance path loss model provide a refined framework for simulating and planning reliable Internet of Things (IoT) deployments in complex indoor scenarios.

### Continuation of the Research and Next Steps

The continuation of this work will focus on enhancing the precision and practical utility of the proposed models by expanding the experimental methodology. The immediate next step is to conduct a complementary measurement campaign employing multiple transceiver positions and other LoRa frequency bands. This will refine the shadowing model and further generalize the path loss parameters (*n*, FAF) across the entire building and operation frequencies, transforming the current baseline into a high-resolution predictive tool. Subsequently, the study will be extended to systematically evaluate the performance trade-offs offered by different LoRa configurations (Spreading Factor and bandwidth). The objective is to derive adaptive transmission strategies that maximize reliability or energy efficiency based on specific sensor application requirements within the smart building.

## Figures and Tables

**Figure 11:**
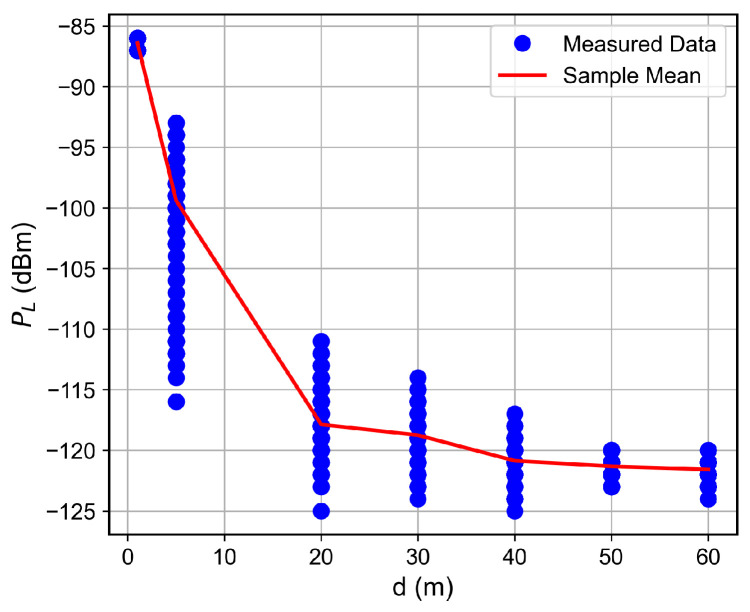
Measured RSSI as a function of the distance for the second floor.

**Figure 12:**
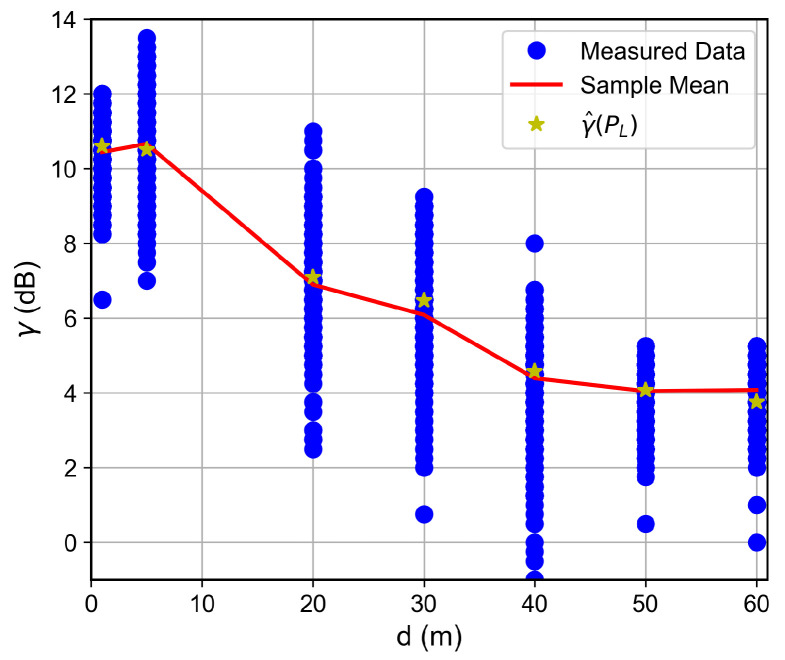
Measured and estimated SNR as a function of the distance for the second floor.

**Figure 13:**
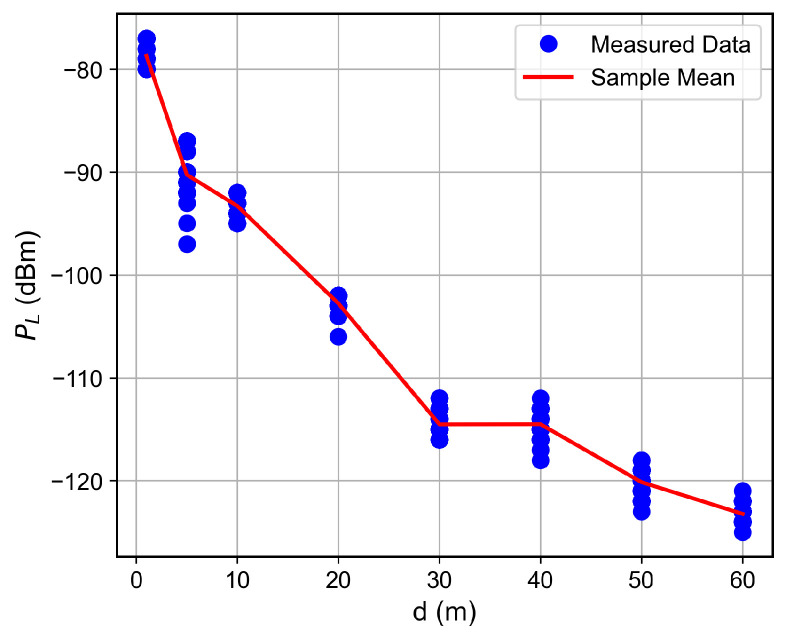
Measured RSSI as a function of the distance for the third floor.

**Figure 14:**
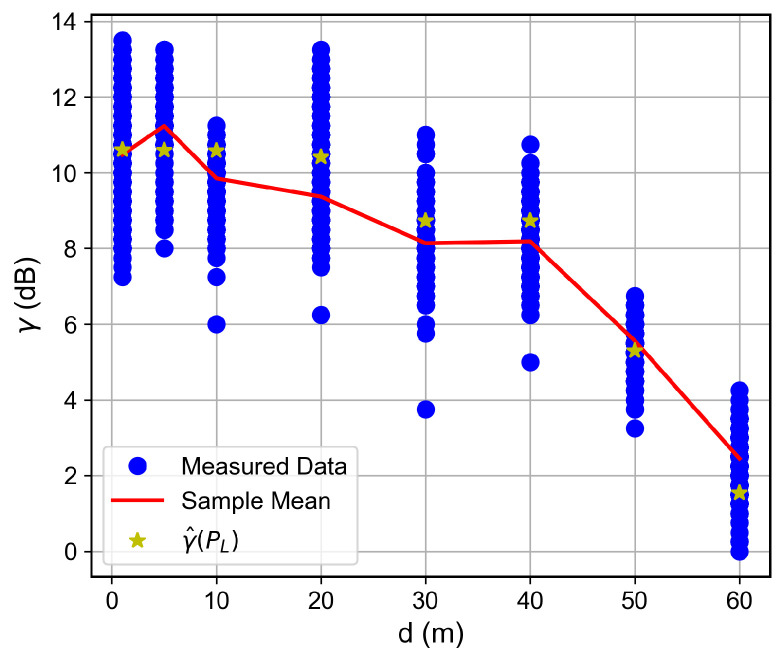
Measured and estimated SNR as a function of the distance for the third floor.

**Table 1:** Physical layer parameters for the measurement configuration.

Parameter	Value
Spreading Factor	12
Signal Bandwidth	125 kHz
Code Rate	4/5
Channel	433 MHz
Cyclic Redundancy Check (CRC)	Activated
Transmission Power	15 dBm

**Table 2:** Mean RSSI (dBm) measurements at 30 m distance for different floor separations, SF, and CR.

Floor	SF	CR 4/5	CR 4/8	Floor Separation
Ground floor	7	−112	−110	3 floors
	9	−107	−110	
	12	−109	−109	
First floor	7	−108	−107	2 floors
	9	−109	−108	
	12	−108	−109	
Second floor	7	−106	−109	1 floor
	9	−105	−110	
	12	−105	−106	
Third floor	7	−98	−95	Same floor
	9	−96	−95	
	12	−96	−97	

## Data Availability

The raw data supporting the conclusions of this article will be made available by the authors upon request.

## Abbreviations

The following abbreviations are used in this manuscript:

CEAR	Center of Alternative and Renewable Energies
Chirp	Compressed High-Intensity Radar Pulses
CR	Code Rate
CRC	Cyclic Redundancy Check
CSS	Chirp Spread Spectrum
DT	Digital Twin
FAF	Floor Attenuation Factor
FSMC	Finite-State Markov Chain
FSPL	Free-Space Path-Loss
IoT	Internet of Things
ISM	Industrial, Scientific and Medical
LFSR	Linear-Feedback Shift Register
LoRa	Long-Range
LoRaWAN	Long Range Wide Area Network
LPWAN	Low Power Wide-Area Network
MAC	Media Access Control
MSSF	Markov Small-Scale Fading
NLoS	Non-Line-of-Sight
PL	Path-Loss
PLR	Packet Loss Rate
PDF	Probability Density Function
RSSI	Receiver Signal Strength Indicator
SF	Spreading Factor
SNR	Signal-to-Noise Ratio
UFPB	Federal University of Paraiba
USB	Universal Serial Bus
Wi-Fi	Wireless Fidelity
